# Di­chlorido­bis­[1-(2,4,6-tri­methyl­phen­yl)-1*H*-imidazole-κ*N* ^3^]copper(II)

**DOI:** 10.1107/S1600536813028821

**Published:** 2013-10-26

**Authors:** Yantao Zhang, Zhuzhen Lin

**Affiliations:** a Health Vocation and Technical College of Guangzhou Medical University, Guangzhou, People’s Republic of China

## Abstract

In the title complex, [CuCl_2_(C_12_H_14_N_2_)_2_], the Cu^2+^ cation is situated on an inversion centre and is coordinated by two N atoms from symmetry-related 1-mesityl-1*H*-imidazole ligands and by two chloride anions in a slightly distorted square-planar geometry. In the organic ligand, the dihedral angle between the benzene ring of the mesityl moiety and the imidazole ring is 76.99 (18)°. Weak intra­molecular C—H⋯Cl hydrogen-bonding inter­actions consolidate the mol­ecular conformation.

## Related literature

For related structures, see: Awwadi (2013 ▶); Jia *et al.* (2005 ▶). For the bioactivity of Cu complexes, see: Beaudoin *et al.* (2009 ▶); Deegana *et al.* (2007 ▶); Pettit & Ueda (1992 ▶). For the photochemistry of Cu complexes, see: Kuang *et al.* (2002 ▶); Raptopoulou *et al.* (1998 ▶); Teyssot *et al.* (2007 ▶).
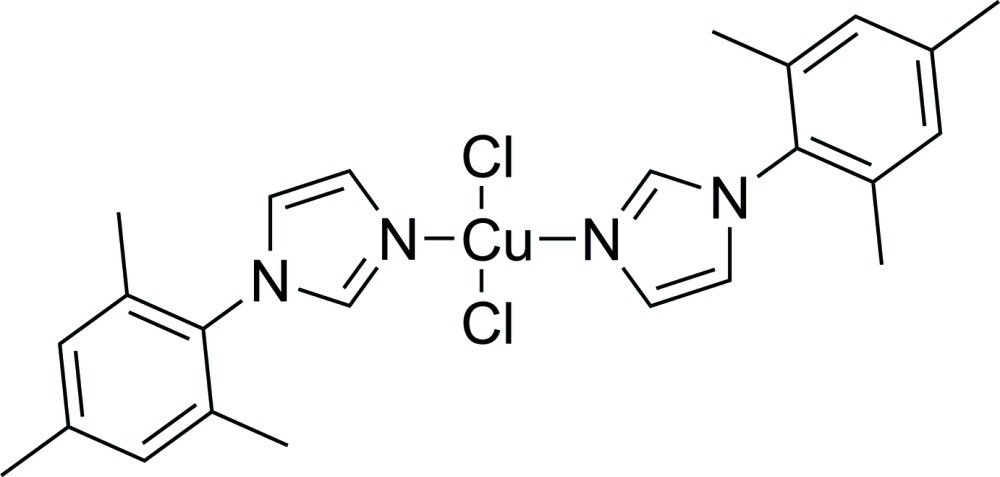


## Experimental

### 

#### Crystal data

[CuCl_2_(C_12_H_14_N_2_)_2_]
*M* *_r_* = 506.94
Monoclinic, 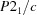

*a* = 7.1488 (6) Å
*b* = 19.7517 (18) Å
*c* = 8.5126 (7) Å
β = 92.674 (8)°
*V* = 1200.68 (18) Å^3^
*Z* = 2
Cu *K*α radiation
μ = 3.47 mm^−1^
*T* = 298 K
0.44 × 0.32 × 0.05 mm

#### Data collection

Bruker APEX CCD diffractometer
Absorption correction: multi-scan (*SADABS*; Bruker, 2008 ▶) *T*_min_ = 0.311, *T*_max_ = 0.846
5702 measured reflections
2114 independent reflections
1738 reflections with *I* > 2σ(*I*)
*R*_int_ = 0.036

#### Refinement

*R*[*F* ^2^ > 2σ(*F* ^2^)] = 0.052
*wR*(*F* ^2^) = 0.145
*S* = 1.02
2114 reflections
145 parameters
H-atom parameters constrained
Δρ_max_ = 1.16 e Å^−3^
Δρ_min_ = −1.18 e Å^−3^

### 

Data collection: *APEX2* (Bruker, 2008 ▶); cell refinement: *SAINT* (Bruker, 2008 ▶); data reduction: *SAINT*; program(s) used to solve structure: *SHELXS97* (Sheldrick, 2008 ▶); program(s) used to refine structure: *SHELXL97* (Sheldrick, 2008 ▶); molecular graphics: *SHELXTL* (Sheldrick, 2008 ▶); software used to prepare material for publication: *publCIF* (Westrip, 2010 ▶).

## Supplementary Material

Crystal structure: contains datablock(s) I, New_Global_Publ_Block. DOI: 10.1107/S1600536813028821/wm2773sup1.cif

Structure factors: contains datablock(s) I. DOI: 10.1107/S1600536813028821/wm2773Isup2.hkl

Additional supplementary materials:  crystallographic information; 3D view; checkCIF report

## Figures and Tables

**Table 1:** Hydrogen-bond geometry (Å, °)

*D*—H⋯*A*	*D*—H	H⋯*A*	*D*⋯*A*	*D*—H⋯*A*
C1—H1⋯Cl1	0.93	2.55	3.060 (4)	115

**Fig. 1.**
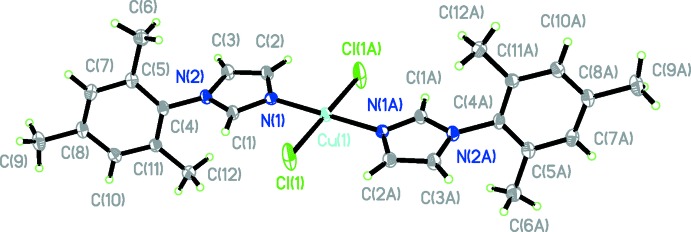
Molecular structure of compound (I). Displacement ellipsoids are shown at the 40% probability level. H atoms are presented as small spheres of arbitrary radius. [Symmetry code A) -x+1, -y+1, -z+1.]

**Fig. 2.**
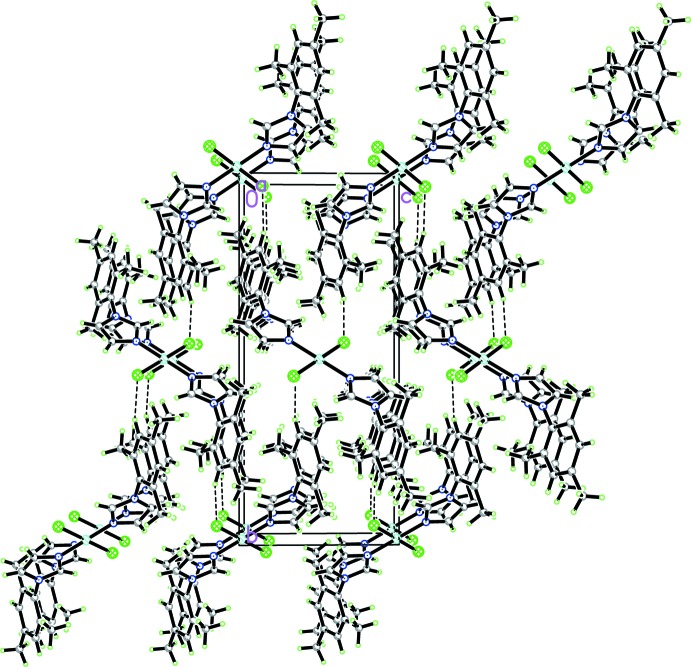
The crystal packing of compound (I). C—H···Cl interactions are shown as dashed lines.

## supplementary crystallographic information

### 1. Comment

In recent years, phosphorescent Cu(II) complexes received attention due to their nontoxic properties (Deegana *et al.*, 2007), which make these complexes applicable in biological probing (Beaudoin *et al.*, 2009; Deegana *et al.*, 2007; Pettit & Ueda, 1992), solar energy conversion, and organic light emitting devices (Kuang *et al.*, 2002; Jia *et al.*, 2005; Teyssot *et al.*, 2007). Our interest is focused on the design and synthesis of phosphorescent Cu(II) complexes with various ancillary ligands, and their applications in anti-cancer therapy (Awwadi, 2013; Raptopoulou *et al.*, 1998). We herein describe the synthesis and structural characterization of the title compound, [CuCl_2_(C_12_H_14_N_2_)_2_], (I).

The molecular structure of compound (I) is shown in Fig. 1. The metal cation is situated on an inversion centre and is coordinated by two N atoms of the 1-mesityl-1H-imidazole ligands and by two chloride anions in a slightly distorted square-planar geometry. The mesityl ring moiety and the imidazole ring are almost orthogonal to each other, with a dihedral angle between the two rings of 76.99 (18) °. Weak intramolecular C—H···Cl hydrogen bonding interactions consolidate the molecular conformation (Fig. 2).

### 2. Experimental

In a Schlenk flask, a solution of 1-mesityl-1H-imidazole (10 ml, 1*M* in CH_2_Cl_2_) was added to a suspension of CuCl_2_ (5 mmol) in 10 ml CH_2_Cl_2_ at room temperature. The reaction was stirred in the absence of light for 6 h at this temperature. The reaction mixture was then filtered in the dark and the volume of the solution reduced to 5.0 ml. Pentane was added to afford the product as an green solid in 40% yield. Single crystals suitable for X-ray diffraction were obtained by slow evaporation of a solution of the title compound in CH_2_Cl_2_ at room temperature.

### 3. Refinement

C-bound H atoms were positioned geometrically and refined as riding atoms, with C—H = 0.93 (aromatic), 0.96 (CH_3_) Å and with *U*_iso_(H)= 1.2 (1.5 for methyl) *U*_eq_(C).

### Crystal data

**Table:**

[CuCl_2_(C_12_H_14_N_2_)_2_]	*F*(000) = 526
*M**_r_* = 506.94	*D*_x_ = 1.402 Mg m^−^^3^
Monoclinic, *P*2_1_/*c*	Cu *K*α radiation, λ = 1.54178 Å
Hall symbol: -P 2ybc	Cell parameters from 7467 reflections
*a* = 7.1488 (6) Å	θ = 2.2–27.0°
*b* = 19.7517 (18) Å	µ = 3.47 mm^−^^1^
*c* = 8.5126 (7) Å	*T* = 298 K
β = 92.674 (8)°	Plate, green
*V* = 1200.68 (18) Å^3^	0.44 × 0.32 × 0.05 mm
*Z* = 2	

### Data collection

**Table:**

Bruker APEX CCD diffractometer	2114 independent reflections
Radiation source: fine-focus sealed tube	1738 reflections with *I* > 2σ(*I*)
Graphite monochromator	*R*_int_ = 0.036
phi and ω scans	θ_max_ = 66.9°, θ_min_ = 4.5°
Absorption correction: multi-scan (*SADABS*; Bruker, 2008)	*h* = −7→8
*T*_min_ = 0.311, *T*_max_ = 0.846	*k* = −21→23
5702 measured reflections	*l* = −10→9

### Refinement

**Table:**

Refinement on *F*^2^	Primary atom site location: structure-invariant direct methods
Least-squares matrix: full	Secondary atom site location: difference Fourier map
*R*[*F*^2^ > 2σ(*F*^2^)] = 0.052	Hydrogen site location: inferred from neighbouring sites
*wR*(*F*^2^) = 0.145	H-atom parameters constrained
*S* = 1.02	*w* = 1/[σ^2^(*F*_o_^2^) + (0.0792*P*)^2^ + 1.6476*P*] where *P* = (*F*_o_^2^ + 2*F*_c_^2^)/3
2114 reflections	(Δ/σ)_max_ < 0.001
145 parameters	Δρ_max_ = 1.16 e Å^−^^3^
0 restraints	Δρ_min_ = −1.18 e Å^−^^3^

### Special details

**Table:**

Geometry. All e.s.d.'s (except the e.s.d. in the dihedral angle between two l.s. planes) are estimated using the full covariance matrix. The cell e.s.d.'s are taken into account individually in the estimation of e.s.d.'s in distances, angles and torsion angles; correlations between e.s.d.'s in cell parameters are only used when they are defined by crystal symmetry. An approximate (isotropic) treatment of cell e.s.d.'s is used for estimating e.s.d.'s involving l.s. planes.
Refinement. Refinement of *F*^2^ against ALL reflections. The weighted *R*-factor *wR* and goodness of fit *S* are based on *F*^2^, conventional *R*-factors *R* are based on *F*, with *F* set to zero for negative *F*^2^. The threshold expression of *F*^2^ > σ(*F*^2^) is used only for calculating *R*-factors(gt) *etc*. and is not relevant to the choice of reflections for refinement. *R*-factors based on *F*^2^ are statistically about twice as large as those based on *F*, and *R*- factors based on ALL data will be even larger.

### Fractional atomic coordinates and isotropic or equivalent isotropic displacement parameters (Å^2^)

**Table:**

	*x*	*y*	*z*	*U*_iso_*/*U*_eq_	
Cu1	0.5000	0.5000	0.5000	0.0301 (3)	
Cl1	0.2765 (2)	0.45641 (8)	0.65130 (12)	0.0843 (6)	
N1	0.3864 (4)	0.45022 (13)	0.3140 (3)	0.0271 (6)	
N2	0.2163 (4)	0.37951 (14)	0.1669 (3)	0.0298 (6)	
C5	−0.0735 (5)	0.33960 (18)	0.0315 (4)	0.0321 (7)	
C7	−0.1896 (5)	0.28533 (19)	−0.0109 (4)	0.0370 (8)	
H7	−0.2996	0.2937	−0.0704	0.044*	
C4	0.0884 (4)	0.32493 (17)	0.1230 (4)	0.0292 (7)	
C1	0.2452 (5)	0.40602 (17)	0.3112 (4)	0.0305 (7)	
H1	0.1762	0.3950	0.3976	0.037*	
C2	0.4488 (5)	0.45081 (18)	0.1632 (4)	0.0348 (8)	
H2	0.5475	0.4769	0.1295	0.042*	
C8	−0.1471 (5)	0.21907 (18)	0.0325 (4)	0.0358 (8)	
C11	0.1388 (5)	0.25940 (17)	0.1680 (4)	0.0296 (7)	
C10	0.0168 (5)	0.20712 (18)	0.1221 (4)	0.0345 (8)	
H10	0.0461	0.1630	0.1525	0.041*	
C12	0.3186 (5)	0.2443 (2)	0.2602 (4)	0.0399 (9)	
H12A	0.3064	0.2570	0.3681	0.060*	
H12B	0.4189	0.2695	0.2171	0.060*	
H12C	0.3452	0.1968	0.2543	0.060*	
C6	−0.1178 (5)	0.41059 (19)	−0.0233 (5)	0.0430 (9)	
H6A	−0.1165	0.4405	0.0657	0.065*	
H6B	−0.2395	0.4114	−0.0760	0.065*	
H6C	−0.0256	0.4251	−0.0946	0.065*	
C9	−0.2722 (6)	0.1608 (2)	−0.0181 (6)	0.0521 (10)	
H9A	−0.2241	0.1196	0.0282	0.078*	
H9B	−0.2757	0.1569	−0.1306	0.078*	
H9C	−0.3965	0.1687	0.0160	0.078*	
C3	0.3455 (5)	0.40795 (19)	0.0722 (4)	0.0372 (8)	
H3	0.3590	0.3993	−0.0341	0.045*	

### Atomic displacement parameters (Å^2^)

**Table:**

	*U*^11^	*U*^22^	*U*^33^	*U*^12^	*U*^13^	*U*^23^
Cu1	0.0369 (4)	0.0328 (4)	0.0208 (4)	−0.0118 (3)	0.0032 (3)	−0.0030 (3)
Cl1	0.1018 (10)	0.1223 (12)	0.0307 (5)	−0.0855 (9)	0.0242 (5)	−0.0249 (6)
N1	0.0296 (14)	0.0288 (14)	0.0230 (13)	−0.0028 (11)	0.0024 (10)	−0.0001 (11)
N2	0.0329 (14)	0.0316 (15)	0.0252 (13)	−0.0094 (12)	0.0043 (11)	−0.0045 (11)
C5	0.0282 (16)	0.0331 (18)	0.0352 (17)	−0.0009 (14)	0.0049 (13)	−0.0074 (15)
C7	0.0271 (17)	0.042 (2)	0.042 (2)	−0.0025 (15)	−0.0027 (14)	−0.0063 (16)
C4	0.0296 (16)	0.0339 (17)	0.0242 (15)	−0.0089 (14)	0.0044 (12)	−0.0074 (14)
C1	0.0339 (17)	0.0350 (17)	0.0229 (15)	−0.0094 (14)	0.0042 (13)	−0.0035 (13)
C2	0.0401 (19)	0.0388 (19)	0.0259 (17)	−0.0128 (15)	0.0050 (14)	−0.0009 (14)
C8	0.0323 (18)	0.0363 (19)	0.0391 (19)	−0.0084 (15)	0.0041 (14)	−0.0085 (15)
C11	0.0331 (17)	0.0327 (18)	0.0234 (16)	−0.0054 (14)	0.0045 (13)	−0.0011 (13)
C10	0.0372 (19)	0.0314 (18)	0.0354 (18)	−0.0055 (15)	0.0057 (14)	−0.0022 (15)
C12	0.042 (2)	0.042 (2)	0.0352 (19)	−0.0074 (16)	−0.0045 (15)	0.0033 (16)
C6	0.038 (2)	0.036 (2)	0.055 (2)	0.0018 (16)	−0.0016 (17)	−0.0028 (17)
C9	0.042 (2)	0.043 (2)	0.071 (3)	−0.0121 (18)	−0.0017 (19)	−0.012 (2)
C3	0.045 (2)	0.046 (2)	0.0213 (16)	−0.0150 (17)	0.0087 (14)	−0.0058 (15)

### Geometric parameters (Å, º)

**Table:**

Cu1—N1^i^	2.004 (3)	C6—H6A	0.9600
Cu1—N1	2.004 (3)	C6—H6B	0.9600
Cu1—Cl1^i^	2.2684 (10)	C6—H6C	0.9600
Cu1—Cl1	2.2684 (10)	C7—C8	1.390 (5)
N1—C1	1.334 (4)	C7—H7	0.9300
N1—C2	1.378 (4)	C8—C10	1.388 (5)
N2—C1	1.343 (4)	C8—C9	1.509 (5)
N2—C3	1.374 (4)	C9—H9A	0.9600
N2—C4	1.451 (4)	C9—H9B	0.9600
C1—H1	0.9300	C9—H9C	0.9600
C2—C3	1.345 (5)	C10—C11	1.396 (5)
C2—H2	0.9300	C10—H10	0.9300
C3—H3	0.9300	C11—C12	1.504 (5)
C4—C11	1.392 (5)	C12—H12A	0.9600
C4—C5	1.395 (5)	C12—H12B	0.9600
C5—C7	1.393 (5)	C12—H12C	0.9600
C5—C6	1.507 (5)		
			
N1^i^—Cu1—N1	180.00 (13)	H6A—C6—H6B	109.5
N1^i^—Cu1—Cl1^i^	89.56 (8)	C5—C6—H6C	109.5
N1—Cu1—Cl1^i^	90.44 (8)	H6A—C6—H6C	109.5
N1^i^—Cu1—Cl1	90.44 (8)	H6B—C6—H6C	109.5
N1—Cu1—Cl1	89.56 (8)	C8—C7—C5	122.4 (3)
Cl1^i^—Cu1—Cl1	180.00 (7)	C8—C7—H7	118.8
C1—N1—C2	105.5 (3)	C5—C7—H7	118.8
C1—N1—Cu1	127.8 (2)	C10—C8—C7	118.3 (3)
C2—N1—Cu1	126.5 (2)	C10—C8—C9	120.1 (3)
C1—N2—C3	107.4 (3)	C7—C8—C9	121.6 (3)
C1—N2—C4	126.4 (3)	C8—C9—H9A	109.5
C3—N2—C4	125.8 (3)	C8—C9—H9B	109.5
N1—C1—N2	110.8 (3)	H9A—C9—H9B	109.5
N1—C1—H1	124.6	C8—C9—H9C	109.5
N2—C1—H1	124.6	H9A—C9—H9C	109.5
C3—C2—N1	109.8 (3)	H9B—C9—H9C	109.5
C3—C2—H2	125.1	C8—C10—C11	121.9 (3)
N1—C2—H2	125.1	C8—C10—H10	119.0
C2—C3—N2	106.6 (3)	C11—C10—H10	119.0
C2—C3—H3	126.7	C4—C11—C10	117.4 (3)
N2—C3—H3	126.7	C4—C11—C12	122.1 (3)
C11—C4—C5	123.0 (3)	C10—C11—C12	120.5 (3)
C11—C4—N2	117.9 (3)	C11—C12—H12A	109.5
C5—C4—N2	119.1 (3)	C11—C12—H12B	109.5
C7—C5—C4	117.0 (3)	H12A—C12—H12B	109.5
C7—C5—C6	121.4 (3)	C11—C12—H12C	109.5
C4—C5—C6	121.5 (3)	H12A—C12—H12C	109.5
C5—C6—H6A	109.5	H12B—C12—H12C	109.5
C5—C6—H6B	109.5		
			
Cl1^i^—Cu1—N1—C1	174.6 (3)	C11—C4—C5—C7	1.9 (5)
Cl1—Cu1—N1—C1	−5.4 (3)	N2—C4—C5—C7	178.4 (3)
Cl1^i^—Cu1—N1—C2	0.1 (3)	C11—C4—C5—C6	−176.2 (3)
Cl1—Cu1—N1—C2	−179.9 (3)	N2—C4—C5—C6	0.3 (5)
C2—N1—C1—N2	−0.3 (4)	C4—C5—C7—C8	−1.1 (5)
Cu1—N1—C1—N2	−175.7 (2)	C6—C5—C7—C8	177.1 (4)
C3—N2—C1—N1	0.1 (4)	C5—C7—C8—C10	0.4 (5)
C4—N2—C1—N1	173.2 (3)	C5—C7—C8—C9	−178.4 (4)
C1—N1—C2—C3	0.4 (4)	C7—C8—C10—C11	−0.5 (5)
Cu1—N1—C2—C3	175.9 (2)	C9—C8—C10—C11	178.3 (3)
N1—C2—C3—N2	−0.3 (4)	C5—C4—C11—C10	−2.0 (5)
C1—N2—C3—C2	0.1 (4)	N2—C4—C11—C10	−178.6 (3)
C4—N2—C3—C2	−173.0 (3)	C5—C4—C11—C12	177.0 (3)
C1—N2—C4—C11	−74.2 (4)	N2—C4—C11—C12	0.4 (5)
C3—N2—C4—C11	97.6 (4)	C8—C10—C11—C4	1.2 (5)
C1—N2—C4—C5	109.1 (4)	C8—C10—C11—C12	−177.8 (3)
C3—N2—C4—C5	−79.1 (4)		

Symmetry code: (i) −*x*+1, −*y*+1, −*z*+1.

### Hydrogen-bond geometry (Å, º)

**Table:**

*D*—H···*A*	*D*—H	H···*A*	*D*···*A*	*D*—H···*A*
C1—H1···Cl1	0.93	2.55	3.060 (4)	115
